# β-Arrestin2 Regulates Lysophosphatidic Acid-Induced Human Breast Tumor Cell Migration and Invasion *via* Rap1 and IQGAP1

**DOI:** 10.1371/journal.pone.0056174

**Published:** 2013-02-06

**Authors:** Mistre Alemayehu, Magdalena Dragan, Cynthia Pape, Iram Siddiqui, David B. Sacks, Gianni M. Di Guglielmo, Andy V. Babwah, Moshmi Bhattacharya

**Affiliations:** 1 Department of Physiology and Pharmacology, Western University, London, Ontario, Canada; 2 Department of Pathology, Western University, London, Ontario, Canada; 3 The Children’s Health Research Institute, Western University, London, Ontario, Canada; 4 Lawson Health Research Institute, Western University, London, Ontario, Canada; 5 Department of Obstetrics and Gynecology, Western University, London, Ontario, Canada; 6 Department of Laboratory Medicine, National Institutes of Health, Bethesda, Maryland, United States of America; Institute of Molecular and Cell Biology, Singapore

## Abstract

β-arrestins play critical roles in chemotaxis and cytoskeletal reorganization downstream of several receptor types, including G protein-coupled receptors (GPCRs), which are targets for greater than 50% of all pharmaceuticals. Among them, receptors for lysophosphatidic acid (LPA), namely LPA_1_ are overexpressed in breast cancer and promote metastatic spread. We have recently reported that β-arrestin2 regulates LPA_1_-mediated breast cancer cell migration and invasion, although the underlying molecular mechanisms are not clearly understood. We show here that LPA induces activity of the small G protein, Rap1 in breast cancer cells in a β-arrestin2-dependent manner, but fails to activate Rap1 in non-malignant mammary epithelial cells. We found that Rap1A mRNA levels are higher in human breast tumors compared to healthy patient samples and Rap1A is robustly expressed in human ductal carcinoma *in situ* and invasive tumors, in contrast to the normal mammary ducts. Rap1A protein expression is also higher in aggressive breast cancer cells (MDA-MB-231 and Hs578t) relative to the weakly invasive MCF-7 cells or non-malignant MCF10A mammary cells. Depletion of Rap1A expression significantly impaired LPA-stimulated migration of breast cancer cells and invasiveness in three-dimensional Matrigel cultures. Furthermore, we found that β-arrestin2 associates with the actin binding protein IQGAP1 in breast cancer cells, and is necessary for the recruitment of IQGAP1 to the leading edge of migratory cells. Depletion of IQGAP1 blocked LPA-stimulated breast cancer cell invasion. Finally, we have identified that LPA enhances the binding of endogenous Rap1A to β-arrestin2, and also stimulates Rap1A and IQGAP1 to associate with LPA_1_. Thus our data establish novel roles for Rap1A and IQGAP1 as critical regulators of LPA-induced breast cancer cell migration and invasion.

## Introduction

Breast cancer is the leading cause of cancer-related deaths in women world-wide and metastasis accounts for the majority of these deaths [1]. Thus, characterization of the signaling mechanisms involved in breast cancer cell migration and invasion, processes that are critically required for the metastatic spread of cancer is crucial for the identification of new therapeutic targets. The β-arrestins (β-arrestin1 and 2) are ubiquitously expressed proteins that are instrumental in attenuating G protein-coupled receptor (GPCR) signaling [2], [3]. β-Arrestins can also function as molecular scaffolds for the organization of signaling complexes and thereby regulate cell migration [2], [4], [5] downstream of various receptors including GPCRs [6]–[9], receptor kinases such as transforming growth factor β receptor-III and insulin-like growth factor-1 receptor [4], [10], [11].

Emerging roles of β-arrestins in tumorigenesis have been demonstrated using *in vitro* [12], [13] and *in vivo* model systems [14]–[16]. β-arrestins can associate with and regulate the oncoprotein Mdm2, a negative regulator of the tumor suppressor p53 [17], [18]. In breast cancer cells, β-arrestins regulate stress fiber formation *via* Rho GTPases [6], or by activating the actin filament-severing protein cofilin [8]. Recently, a direct role for β-arrestins in regulating breast cancer metastasis has been demonstrated using a xenograft model with MDA-MB-231 cells [19]. We have previously reported that β-arrestin2 mRNA levels are elevated in patient breast tumors samples at advanced stages of the disease [20]. Consistent with these observations, a recent study has shown that β-arrestin2 protein expression increased with the progression of breast cancer invasiveness [21]. Furthermore, we found that β-arrestins regulate breast cancer invasion by regulating the activity of matrix metalloproteases [20], [22]. Although β-arrestin2 has been suggested to regulate actin cytoskeleton organization by acting as scaffolds for actin binding proteins [5], [23] and thereby regulate cell motility and cancer invasion, the underlying molecular mechanism by which β-arrestins regulate cancer cell migration and invasion remain largely unknown.

β-Arrestins regulate internalization of LPA_1_, a member of the endothelial differentiation gene (EDG2) [24]. LPA_1_ binds lysophosphatidic acid (LPA), a blood-borne chemoattractant mitogenic lipid molecule that regulates actin cytoskeletal dynamics necessary for cell migration and thus plays important roles in processes such as wound healing [25]. An LPA_1_ antagonist is currently in clinical trials for fibrotic disease (http://www.amirapharm.com). LPA has emerging roles in cancer and can mediate the ‘hallmarks of cancer’. LPA induces the transformation of benign cells into invasive carcinoma cells, stimulates tumor growth, angiogenesis, invasion, and metastasis [26]. Autotaxin, a key enzyme in LPA production in blood, is overexpressed in various human malignancies, including breast cancer [26], [27]. LPA signaling *via* LPA_1_, stimulates proliferation and metastasis of MDA-MB-231 breast cancer cells in a xenograft mouse model [28]. Although LPA_1_ is present in normal mammary epithelial cells, it is aberrantly expressed in breast cancer [20], [29]. Additionally, expression of LPA_1_ in mammary epithelial cells of transgenic mice promotes the development of metastatic mammary tumors [29].

A few studies have shed light on the mechanisms by which LPA_1_ regulates breast cancer cell migration, implicating a role for phosphatidylinositol 3-kinases/PAK1/ERK signaling [30] and Rho kinases [31]. However, we have shown a major role for G_i/o_ signaling pathways in LPA_1_-stimulated breast cancer cell migration and invasion signaling *via* β-arrestins and Ral GTPases [20]. We found that LPA_1_ is overexpressed in patient breast tumors [20] and high levels of LPA_1_ transcripts have been found in numerous breast cancer cell lines [31]. We showed that LPA_1_ activation induces migration and invasion of breast cancer cells *via* β-arrestin2 and the small GTPase Ral [20]. Ral activity can be regulated by its guanine nucleotide exchange factors (GEFs) such as RalGDS as well as β-arrestins in a Ras-*independent* manner [20], [32]. RalGDS can also associate with Rap1A GTPases with 100-times more affinity than Ras [33]. Although Rap1 (composed of A and B subtypes) was originally discovered as a revertant of *k-Ras*-mediated cell transformation, several studies have since identified Ras-*independent* Rap1 signaling involved in various cellular processes including gene transcription, proliferation and growth, adhesion, polarity and migration [34]–[36]. In the past decade, a role for Rap1 and its regulators has emerged in various cancers including breast cancer [34], [37], [38]. However, whether or not LPA signals via Rap1 in breast cancer cells is unknown.

Here we show that Rap1A is aberrantly expressed in human breast tumors. We identify Rap1A as a novel regulator of LPA-induced breast cancer chemotaxis and invasion, and show that LPA stimulates Rap1 activity in breast cancer cells downstream of β-arrestin2, but fails to activate Rap1 in non-malignant mammary epithelial cells. We identify Rap1A and the actin-binding protein IQGAP1 as novel binding partners for β-arrestin2 as well as LPA_1_. IQGAP1 regulates directional cell migration and the establishment of polarized cell morphology [39]–[41]. We also found that β-arrestin2 is co-localized with IQGAP1 in lamellipodia of migratory breast cancer cells in response to LPA, and appears to critically regulate the recruitment of IQGAP1 to the leading edge. Thus, our data establish novel roles for Rap1A and IQGAP1, downstream of β-arrestin2 as critical regulators of LPA-induced breast cancer cell migration and invasion.

## Results

### Rap1A is aberrantly expressed in human breast tumor tissue

**Figure 1:**
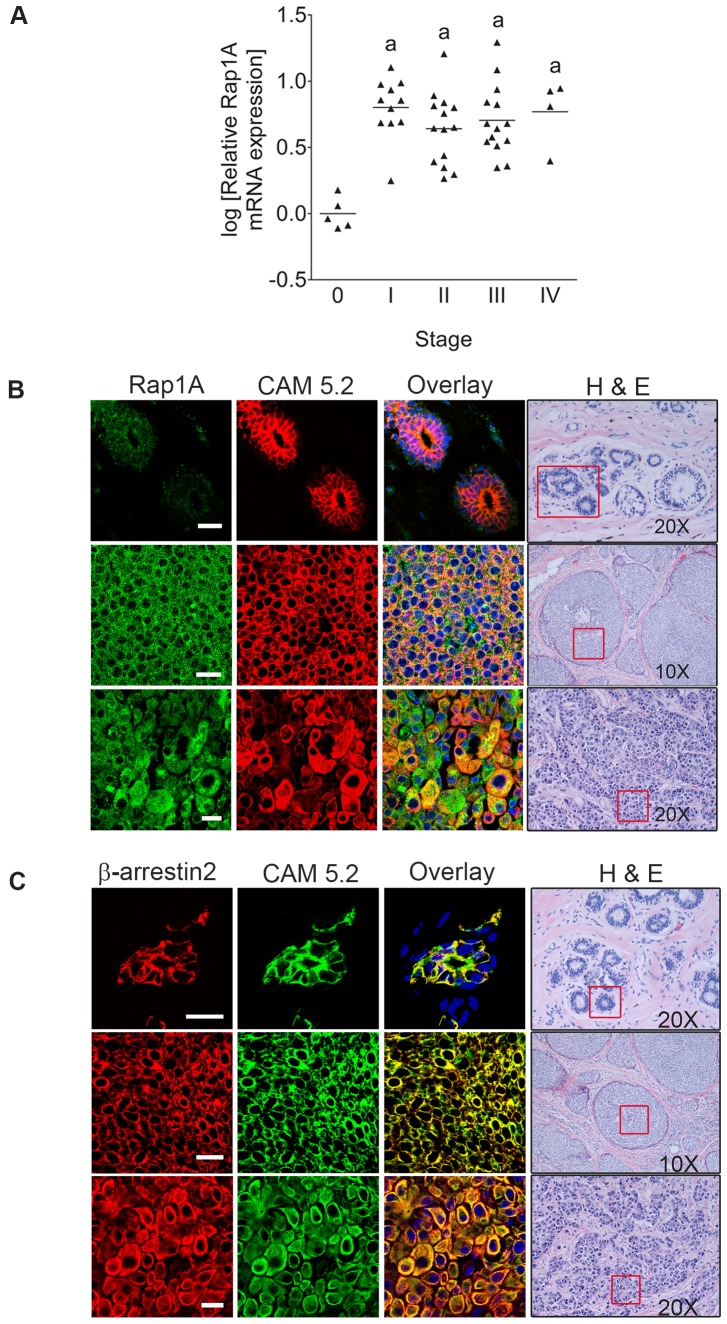
Rap1A is abundantly expressed in human breast tumors. (A) Patient breast tumors from Stages I–IV of the disease express higher levels of Rap1A mRNA compared to non-diseased Stage 0 samples, as determined by quantitative real-time PCR. Data points shown are log-transformed values of relative mRNA expressions compared to average expression in Stage 0 samples. Levels of β-actin were used by the manufacturer to pre-normalize samples. One-way ANOVA statistical analysis was performed on the log-transformed relative expression values and a Dunnett’s multiple comparison post-hoc test was conducted. a, p<0.01, compared to Stage 0. (B) Immunofluorescence and confocal microscopy on formalin-fixed and paraffin-embedded human tissue samples using (B) a rabbit anti-Rap1A polyclonal antibody followed by Alexa-Fluor 488 secondary antibody (green) and (C) a goat anti-β-arrestin2 polyclonal antibody followed by Alexa-Fluor 555 secondary antibody. In order to distinguish cells of epithelial origin, staining for cytokeratin CAM 5.2 was performed using a mouse monoclonal anti-CAM 5.2 antibody (red). Hematoxylin and eosin staining of normal and tumor sections from each group were examined (representative red boxes). Images are representative of four independent experiments. Scale bar, 20 µm.

We have previously shown that the mRNA levels of β-arrestin2 and Ral GTPases are elevated in human breast tumors compared to non-malignant tissue and that these molecules regulate breast cancer migration and invasion [20]. Since the Ral guanine exchange factor, RalGDS, strongly associates with β-arrestins [32] and Rap1A GTPases [33], we determined if the expression of Rap1A was also altered in patient breast tumors. In order to quantify Rap1A expression *in vivo*, Rap1A mRNA levels were measured in an array of 48 cDNA samples derived from breast cancer patients at Stages 0-IV of the disease. We found significantly higher Rap1A mRNA levels in Stage I–IV samples relative to Stage 0 (pathology-confirmed normal) breast tissue (Figure 1A).

We also performed immunohistochemical analyses in a panel of normal, ductal carcinoma *in situ* (DCIS), and invasive human mammary tissue samples. We observed that in normal ductal cells, which strongly displayed the epithelial marker cytokeratin CAM5.2, contained weak Rap1A expression. The relatively little Rap1A expression was detected in the epithelial tissue and absent from the stromal mammary tissue (Figure 1B). However, Rap1A was robustly expressed in DCIS lesions and invasive cancer. We have previously shown that the mRNA levels of β-arrestin2 and Ral GTPases are high in human breast tumors that these molecules regulate breast cancer migration and invasion [20]. We also performed immunohistochemistical analysis to look at the localization of β-arrestin2 in normal and tumor tissue. We observed that normal ductal cells, which strongly displayed the epithelial marker cytokeratin CAM5.2 also expressed β-arrestin2 (Figure 1C). We found that similar to Rap1A, β-arrestin2 was also present in DCIS and invasive infiltrating ductal carcinoma (Figure 1C). These results therefore show that β-arrestins, but not Rap1 GTPases, are strongly expressed in normal mammary duct, and that both Rap1 and β-arrestins are intensely expressed in DCIS and invasive tumors.

### Rap1A is aberrantly expressed in breast cancer cell lines and regulates invasiveness

**Figure 2:**
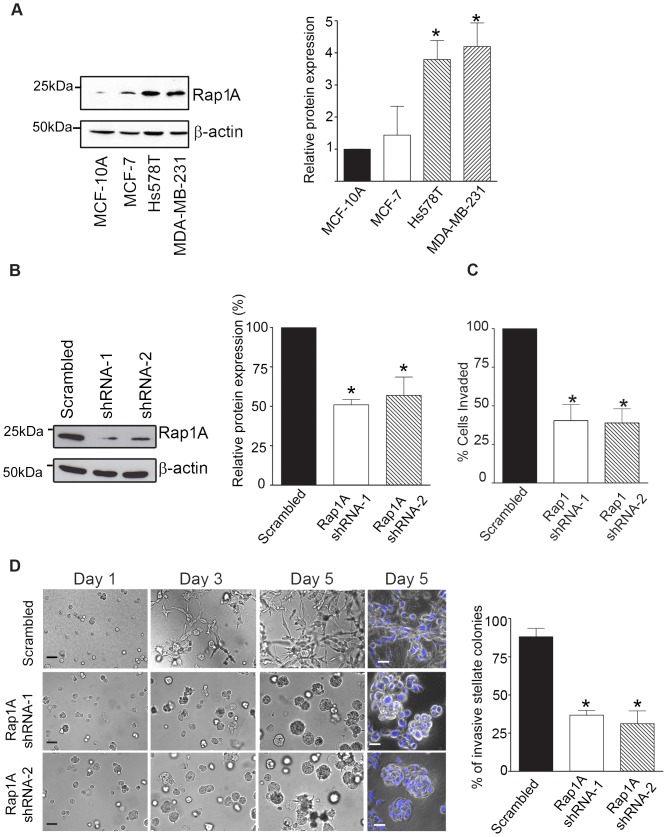
Rap1A is abundantly expressed in invasive human breast cancer cells and regulates invasiveness. (A) The aggressive breast cancer cell lines MDA-MB-231 and Hs578T express elevated levels of Rap1A protein relative to the non-invasive MCF-7 cells or the non-malignant MCF-10A mammary epithelial cells. Endogenous expression of Rap1A in each cell line was determined by Western blot analysis. A representative blot from five separate experiments is shown. In the graphs, bars represent relative expression to MCF-10A cells. Error bar, SEM *, p<0.05. (B) Knockdown of Rap1A protein expression in MDA-MB-231 cells stably expressing two separate Rap1A shRNA constructs was verified by Western blot analysis. Densitometric analysis shows significantly lower levels of Rap1A expression in Rap1A shRNA expressing cells. *, p<0.05. (C) Knock-down of endogenous Rap1A in serum-starved MDA-MB-231 cells inhibits LPA (10 µM) stimulated cell invasion as assessed using Transwell chamber (Matrigel) invasion assays. (D) MDA-MB-231 cells expressing Rap1A shRNA and suspendend in Matrigel for 5 days display reduced growth of invasive stellate structures relative to cells expressing scrambled control. Nuclei were stained using Hoechst 33258 and visualized by confocal microcopy (blue, last column of panel). Images are representative of five independent experiments, scale bar  =  40 µm. Shape of the colonies was scored as either stellate or spheroidal and the number of stellate colonies is expressed as percentage of the total number of colonies. *, p<0.05 compared to formation of stellate structures by MDA-MB-231 cells expressing scrambled shRNA.

We next determined whether or not Rap1A proteins were altered in three well-established human breast cancer cell lines (MCF-7, MDA-MB-231, and Hs578T) compared to non-malignant mammary epithelial (MCF-10A) cells. Western blot analysis revealed significantly higher levels of Rap1A in the invasive breast cancer Hs578T and MDA-MB-231, compared to the non-invasive MCF-7 cells or the non-tumorigenic MCF-10A cells (Figure 2A, *, p<0.05), thus revealing that Rap1A expression is dysregulated in the aggressive breast cancer cells.

Although Rap1 is a key regulator of E-cadherin [36] and has well-established roles in cell adhesion, its role in breast cancer invasiveness is unknown. In order to determine if Rap1A signaling is involved in LPA-mediated breast cancer cell invasion, we depleted Rap1A from the highly aggressive breast cancer MDA-MB-231 cells by stably expressing two individual Rap1A shRNA constructs (shRNA-1 and shRNA-2). The knockdown of Rap1A protein was verified by Western blot analysis (Figure 2B). Transwell chamber Matrigel-invasion assays showed that depletion of Rap1A significantly reduced cell invasion towards LPA (Figure 2C). The effects of Rap1A knockdown on breast cancer cell invasion was also examined using rigid three-dimensional (3D) cell invasion assays using a reconstituted extracellular matrix (Matrigel) that mimics the *in vivo* micro-environment [20]. Although cell viability was not affected by the depletion of Rap1A (Figure S1), we observed a marked reduction in the number of invasive stellate colonies (Figure 2D). These cells formed spherical organoids (spheroids) that were composed of multiple intact nuclei (Figure 2D, last column of panel), similar to those displayed by non-malignant MCF-10A cells [20]. These results indicate that Rap1A regulates breast cancer cell invasiveness.

### Rap1A interacts with LPA_1_ and regulates LPA-induced breast cancer cell migration

Having observed that Rap1A depletion inhibited invasive properties of tumor cells, we next assessed if the depletion of endogenous Rap1A also blocked LPA-stimulated MDA-MB-231 cell migration. Since LPA is a major constituent of serum, all experiments in this study were done with cells serum-starved for at least 4h. We have previously shown that LPA signaling *via* pertussis-toxin sensitive G proteins stimulates MDA-MB-231 cell migration and invasion [20]. Additionally, dose-response studies indicated that LPA-mediated migration of MDA-MB-231 began to plateau at the 10 µM LPA concentration and thus all experiments were conducted using this concentration of LPA, as reported in other studies [42]–[44].

**Figure 3:**
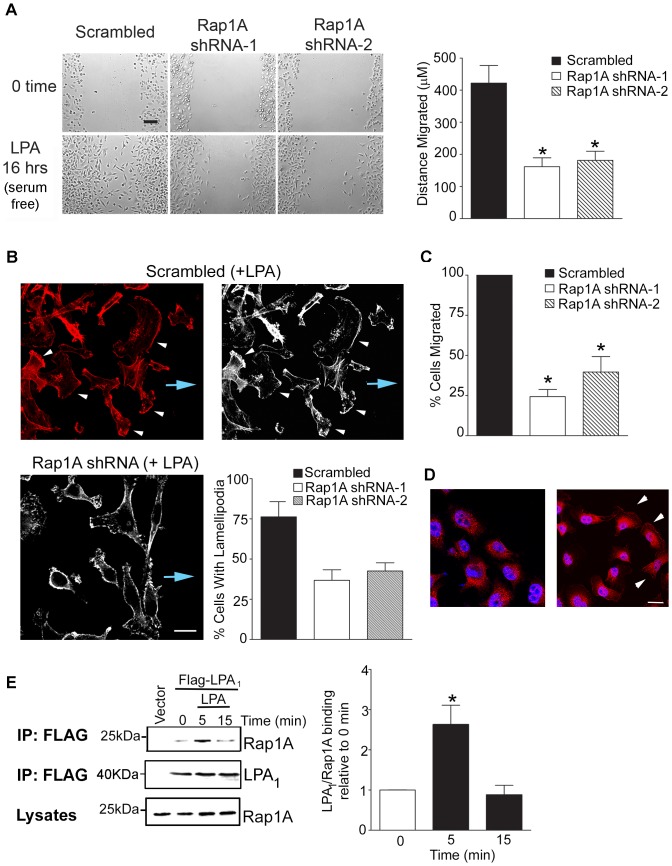
Rap1A associates with LPA_1_ and regulates breast cancer cell migration. (A) Depletion of Rap1A expression significantly blocks scratch closure of MDA-MB-231 cells in response to LPA treatment. Cells were grown to confluence, serum starved for four hours and scratched with a sterile pipette tip. Serum-free medium containing 10 µM LPA was added to the cells which allowed to migrate into the scratch for 16 hours and visualized using an IX-81 Olympus microscope. Cell migration was quantitated using ImagePro software and bars represent the mean distance travelled (in µm) after 16 hours of culturing in LPA. Data from four experments.*, p<0.05, scale bar  =  100 µm. (B) LPA (10 µM) stimulates lamellipodia formation at the leading edge of motile MDA-MB-231cells expressing scrambled shRNA (control, upper panels) in a scratch assay. The effect of depleting endogenous Rap1A from LPA-stimulated cells is shown (lower panel). Cells, grown to confluency were serum starved for 4h and scratched with a sterile pipette tip. Cells were then placed in media containing LPA and allowed to migrate into the scratch for 4 hours, fixed, permeabilized, immunostained and visualized using confocal microscopy. Alexa-Fluor 555 conjugated phalloidin (red) was used to stain F-actin. Loss of color (grayscale image) shows the lamellipodia at the leading edge (white arrowheads). Direction of cell migration shown by blue arrow. Scale bar, 20 µm. Quantitation of cells with lamellipodia was carried out as described under “Materials and Methods” and graphed. Error bar, SEM *, p<0.05. Data from three independent experiments. (C) Migration of serum-starved, Rap1A-depleted MDA-MB-231 cells towards 10 µM LPA is significantly inhibited, as determined by Transwell chamber assays. *, p<0.05. Data from four experments. (D) Confocal micrographs showing the localization of LPA_1_ in MDA-MB-231 cells stably expressing FLAG-LPA_1_ subjected to a scratch assay, as described above. Immunostaining for FLAG (red) with a rabbit anti-FLAG antibody; nuclei were stained using Hoechst 33258. (E) LPA_1_ interacts with endogenous Rap1A. Co-immunoprecipitation studies were performed using serum-starved (4h) cells stably expressing FLAG-LPA_1_ and using a mouse anti-FLAG monoclonal antibody. Endogenous Rap1A was detected by a rabbit polyclonal anti-Rap1A antibody. A representative blot from three independent experiments is shown. Graph illustrates densitometric analysis of blots; data are expressed relative to unstimulated cells. Error bar, SEM *, p<0.05.

Using a scratch assay, we found that Rap1A is necessary for LPA-induced cell motility since loss of Rap1A inhibited scratch closure by with LPA (Figure 3A, and movie S1). Because we found that Rap1A regulated LPA-stimulated directional cell migration, we examined if Rap1A expression was required for LPA-stimulated lamellipodia formation, using a scratch assay to observe polarized cell migration. LPA stimulated lamellipodia formation that were positive for F-actin (Figure 3B, upper panel). Breast cancer cells stably expressing Rap1A shRNA showed a pronounced reduction of LPA-stimulated lamellipodia formation in cells at the leading edge (Figure 3B, lower panel) compared to cells expressing scrambled shRNA (Figure 3B, upper panel). Furthermore, depletion of Rap1A also inhibited LPA-induced cell migration assessed using Transwell chamber assays (Figure 3C). However, we did not observe any effect of Rap1A depletion on cell adhesion (Figure S2). These results reveal that Rap1A is required for LPA stimulated directional migration. We also examined the spatial localization of LPA_1_ in migratory cells. For this purpose, we generated MDA-MB-231 cell lines stably expressing FLAG-LPA_1_ as we have previously described [20], and receptors were detected by immunostaining using an anti-FLAG antibody. We found that in unstimulated cells, FLAG-LPA_1_ is primarily localized intracellularly, as we have previously reported [20] (Figure 3D, left panel **)**. However, upon LPA stimulation, we observed the in addition to being localized intracellularly, LPA_1_ re-localized to the plasma membrane, and also at the leading edge of migratory MDA-MB-231 cells (Figure 3D, right panel **)**.

We next sought to determine whether Rap1 associates with LPA_1_ receptor, to mediate LPA signaling. Using co-immunoprecipitation studies, we found that endogenous Rap1 weakly associated with LPA_1_ under basal conditions. Upon 5 min of LPA stimulation, the interaction was seen to more than double and then returned to background levels by 15 min after stimulation (Figure 3D). Taken together, these results suggest that Rap1 associates with LPA_1_ to regulate LPA-induced breast cancer cell migration.

### LPA activates Rap1 in breast cancer cells but not in non-malignant mammary cells

**Figure 4:**
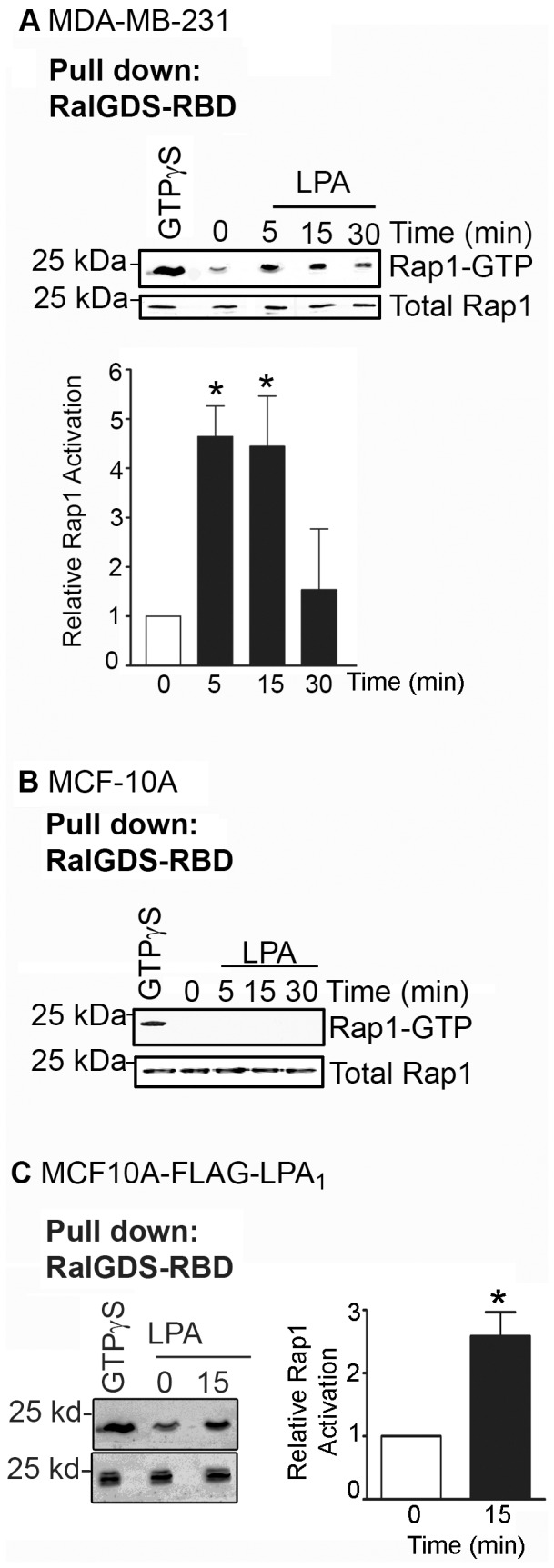
LPA stimulates Rap1 activity in breast cancer cells but not in non-malignant cells. MDA-MB-231 breast cancer cells were serum-starved for 4 hours and stimulated with 10 µM LPA for the indicated times. Cells were then lysed and activated Rap1 was pulled down using RalGDS-RBD-conjugated beads and immunoblotted using a rabbit anti-Rap1 antibody. A representative blot from four independent experiments is shown. (B) MCF-10A cells were stimulated with 10 µM LPA for the indicated times and Rap1 activation was determined. Blot is representative of three independent experiments. (C) MCF-10A cells stably expressing FLAG-LPA_1_ were treated for 15 minutes with 10 µM LPA and subjected to Rap1 activation assay. Blot is representative of three independent experiments.

Although LPA has been shown to activate Rap1 in fibroblasts [45] and endothelial cells [46], whether or not this occurs in any cancer cell types has not been shown. We thus examined if LPA could stimulate Rap1 activity in breast cancer cells. MDA-MB-231 cells were treated with 10 µM LPA, as we previously determined from dose-response studies [20]; this concentration of LPA has also been used by other investigators [43], [44]. All assays were done using serum starved cells to eliminate the effects of LPA present in serum. The content of active (GTP-bound) Rap1 was measured through its ability to strongly bind to its effector protein RalGDS, specifically the Rap1-binding domain of RalGDS (RalGDS-RBD). LPA treatment of MDA-MB-231 cells led to increased Rap1 activity within 5 min of stimulation, and diminished within 30 min (Figure 4A). However, LPA failed to activate Rap1 in the non-tumorigenic MCF-10A cells (Figure 4B), suggesting that LPA may only induce Rap1 activity selectively in breast cancer cells. We have previously shown that MDA-MB-231 cells express significantly higher levels of LPA_1_ than MCF-10A cells [20]. To determine if the lack of LPA_1_ in MCF-10A cells is responsible for the inability of LPA to stimulate Rap1, we examined Rap1 activity in LPA-treated MCF-10A cells stably expressing LPA_1_. We found that LPA did indeed induce Rap1 activity in MCF-10A cells expressing FLAG-LPA_1_ (Figure 4C); we have previously shown that expression of LPA_1_ in the non-malignant MCF-10A cells stimulates these cells to invade in 3D cultures [20].

### Knockdown of β-arrestin2 blocks LPA-stimulated Rap1 activity in breast cancer cells

**Figure 5:**
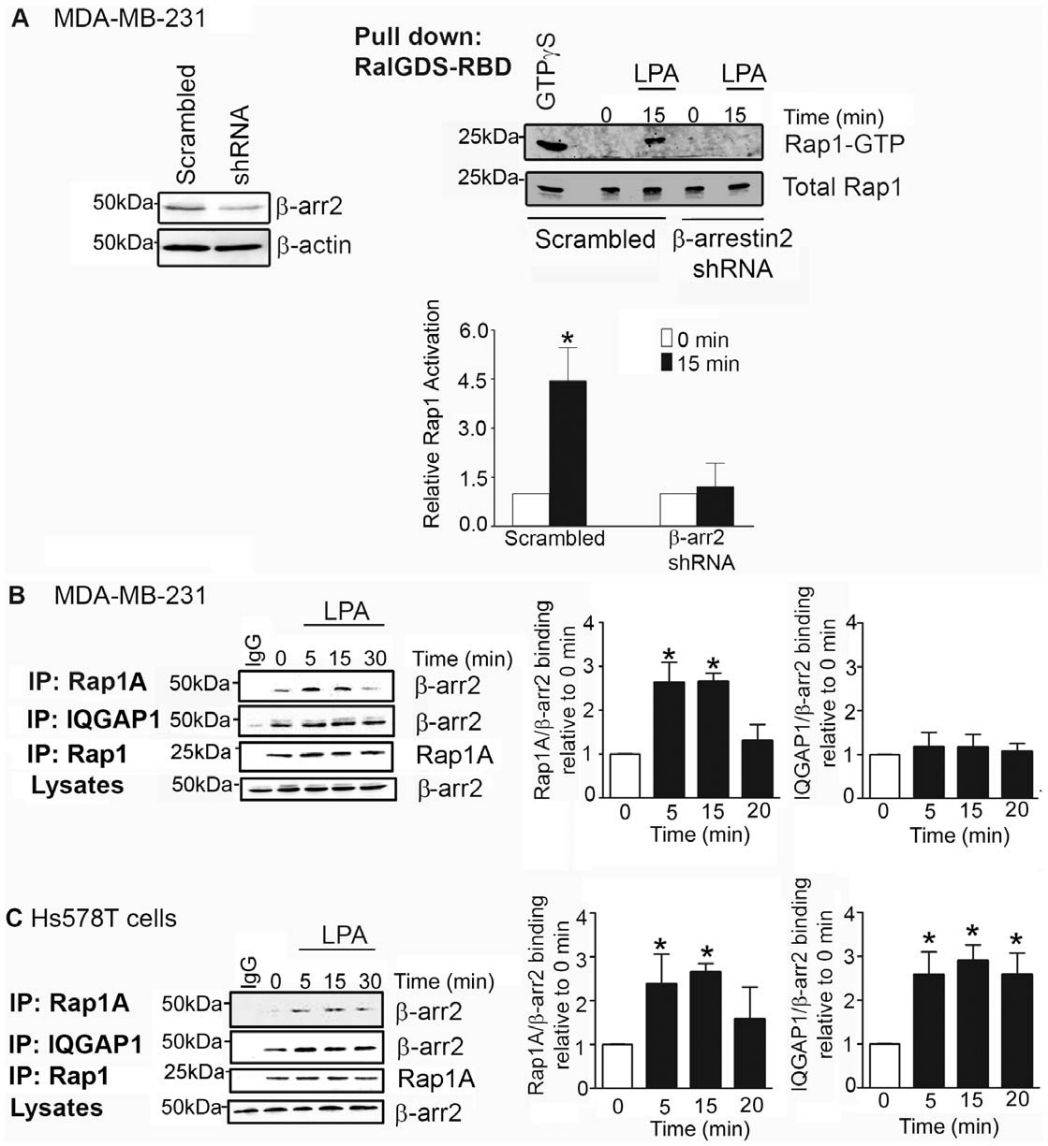
β-arrestin2 regulates Rap1 activity and associates with Rap1A and IQGAP1 in breast cancer cells. (A) Depletion of β-arrestin2 in breast cancer cells blocks LPA-induced Rap1 activation. MDA-MB-231 cells stably expressing either scrambled shRNA or β-arrestin2-targeting shRNA were serum starved for 4 hours and stimulated with 10 µM LPA. Cells were lysed and activated Rap1 was pulled down. Knockdown of β-arrestin2 in MDA-MB-231 cells stably expressing shRNA against β-arrestin2 was verified by Western blot analysis. Representative blots from four independent experiments are shown. (B) The association of endogenous proteins, β-arrestin2, Rap1A and IQGAP1, in breast cancer cells. MDA-MB-231 cells were serum-starved for 4 hours and stimulated with LPA in serum-free media (10 µM). Isolated lysates were subjected to co-immunoprecipitation assays using a rabbit anti-Rap1A antibody or rabbit anti-IQGAP1 antibody and immunoblotted using a monoclonal anti-β-arrestin2 antibody. A representative blot from four independent experiments is shown. Graph illustrates densitometric analysis of blots; data are expressed relative to unstimulated cells. Error bar, SEM *, p<0.05. (C) Endogenous proteins, β-arrestin2, Rap1A and IQGAP1 association in Hs578T cells as described in (B). A representative blot from three independent experiments is shown.

β-arrestin signals *via* Ral GTPases to mediate breast cancer cell invasiveness [20]. Since RalGDS, a RalGEF and a direct binding partner for β-arrestin2 [32], strongly binds to active Rap1 [33], we examined if β-arrestin regulates LPA-induced Rap1 activation. For these studies, we employed a β-arrestin2 shRNA MDA-MB-231 cell line exhibiting reduced β-arrestin2 levels that results in impaired LPA-induced migration and invasion [20]. Here we observed that while LPA significantly induced Rap1 activation in control cells expressing scrambled shRNA sequences, LPA failed to alter Rap1 activity in cells where β-arrestin2 levels were reduced (Figure 5A). These results indicate that β-arrestin2 is required for LPA-mediated Rap1 activation in breast cancer cells.

### Rap1A and β-arrestin2 associate with IQGAP1 in breast cancer cells

We next examined if β-arrestin2 associates with Rap1 to regulate its activity. Using co-immunoprecipitation studies, we found that LPA stimulated the endogenous association of Rap1 with β-arrestin2 after 5–15 min which diminished to basal levels within 30 min of stimulation (Figure 5B). Rap1A has been shown to interact directly with the actin-binding protein, IQGAP1, which has established roles in breast cancer cell migration and invasion [40], [47]. We therefore tested if IQGAP1 was part of the β-arrestin2/Rap1A complex and found that β-arrestin2 did in fact associate with endogenous Rap1A and IQGAP1 in MDA-MB-231 cells (Figure 5B). Addition of LPA modulated only the interaction between β-arrestin2 and Rap1A and did not significantly alter the level of association between β-arrestin2 and IQGAP1. Furthermore, we observed that β-arrestin2 associates with endogenous Rap1A upon LPA stimulation in Hs578T breast cancer cells (Figure 5C). We also observed that endogenous β-arrestin2 binds IQGAP1 under basal conditions in Hs578T cells. In contrast to MDA-MB-231 cells, treatment with LPA further increased the interaction between β-arrestin2 and IQGAP1 and this association persisted even 30 min post stimulation (Figure 5C). The differences in the time frame of association between these signaling molecules might be due to the differences in their expression levels in these two cell lines. Hs578T cells express less β-arrestin2 [20] compared to MDA-MB-231 cells. Overall, these data suggest that the interaction among these endogenous proteins, β-arrestin2, Rap1A and IQGAP1 is not breast cancer cell-type specific

### IQGAP1 associates with LPA­_1_ and regulates LPA-stimulated breast cancer cell invasion

**Figure 6:**
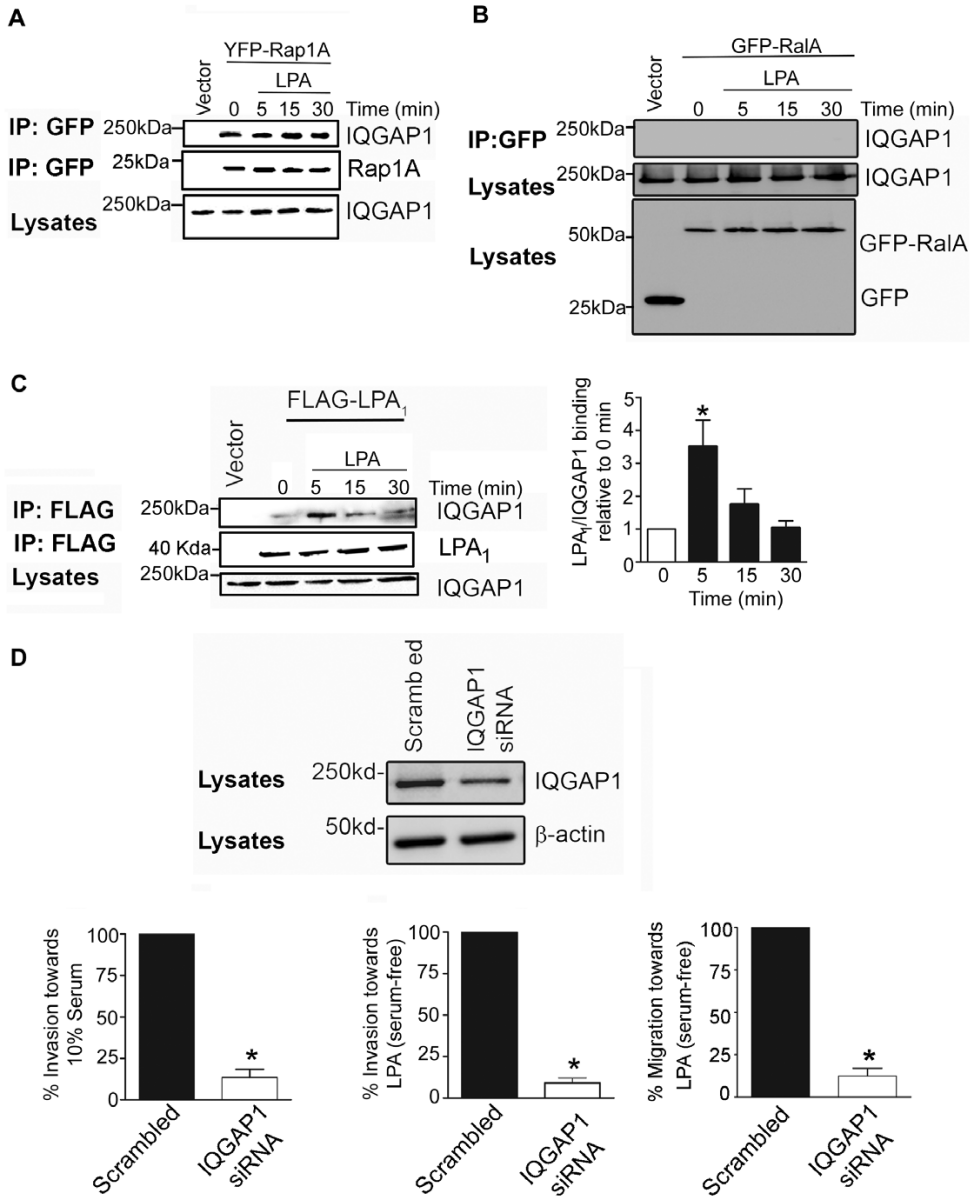
IQGAP1 associates with Rap1A and LPA_1_ and regulates LPA-induced breast cancer cell invasion. (A) Endogenous IQGAP1 binds Rap1A in MDA-MB-231 breast cancer cells. Serum-starved (4h) cells stably expressing YFP-Rap1A or GFP vector control were stimulated with LPA (10 µM) and lysates were subjected to immunoprecipitation assays with an anti-GFP monoclonal antibody and immunoblotted with a rabbit anti-IQGAP1 polyclonal antibody. (B) IQGAP1 does not associate with RalA in MDA-MB-231. Serum starved (4h) cells were treated with 10 µM LPA and co-immunoprecipitation studies done with an anti-GFP monoclonal antibody. Endogenous IQGAP1 was detected using a rabbit anti-IQGAP1 antibody. (C) LPA_1_ interacts with endogenous IQGAP1. Co-immunoprecipitation studies were performed using serum-starved (4h) cells stably expressing FLAG-LPA_1_ and using a mouse anti-FLAG monoclonal antibody. Endogenous IQGAP1 was detected by a rabbit polyclonal anti-IQGAP1 antibody. (D) Depletion of IQGAP1 blocks LPA-induced cell migration and invasion. Serum starved (4h) MDA-MB-231 cells expressing IQGAP1 siRNA were subjected to Transwell migration or Matrigel-invasion assays towards 10 µM LPA (in serum-free media)**.** Results represent data from three independent experiments. *, p<0.05. IQGAP1 expression in MDA-MB-231 cells stably expressing siRNA against IQGAP1 was verified by Western blot analysis. A representative blot from three independent experiments is shown.

Although it has been demonstrated that IQGAP1 directly interacts with Rap1A *in vitro*, using purified proteins [47], whether or not this association occurs in breast cancer cells is not known. Thus we next sought to determine whether or not Rap1A associates with IQGAP1. We found that endogenous IQGAP1 associated constitutively with YFP-Rap1A in MDA-MB-231 cells, and this interaction was not modulated by LPA (Figure 6A). Since IQGAP1 has been shown to associate with various small GTPases including cdc42 and Rac1 [48], we examined whether or not RalA, previously shown to signal downstream of β-arrestin in cancer cells [20], can associate with IQGAP1. We found that unlike Rap1A, RalA did not co-immunoprecipitate with endogenous IQGAP1 from MDA-MB-231 cells (Figure 6B), although RalA was present in immunoprecipitates (data not shown), indicating that the interaction of IQGAP1 is specific to a subset of small GTPases.

We next sought to determine whether or not IQGAP1 binds LPA_1_ and plays a role in transducing its signals to the cytoskeleton to thereby stimulate invasion. We found that endogenous IQGAP1 weakly associated with FLAG-LPA_1_ in MDA-MB-231 cells and this interaction increased significantly upon treatment of cells with LPA for 5 min (Figure 6C). The interaction between IQGAP1 and the receptor decreased within 15 min of stimulation, approaching basal levels after 30 min of LPA stimulation.

To determine a role for IQGAP1 in LPA-induced breast cancer invasiveness, IQGAP1 expression was stably depleted in MDA-MB-231 cells which express the highest level of this protein [49] using IQGAP1 siRNA constructs [40] (Figure 6D). We found that depletion of IQGAP1 from MDA-MB-231 cells significantly blocked serum-induced invasion compared to cells expressing scrambled controls (Figure 6D). Furthermore, diminished levels of IQGAP1 in MDA-MB-231 cells abrogated invasion and migration of breast cancer cells towards LPA, in the absence of serum, compared to cells expressing scrambled controls (Figure 6D). These results implicate IQGAP1 as a novel regulator of LPA-mediated breast cancer cell invasion.

### β-Arrestin2 depletion disrupts LPA-stimulated lamellipodia formation and IQGAP1 localization at the leading edge

Overall, our data indicate that β-arrestin2 scaffolds IQGAP1 and Rap1A in breast cancer cells and that the β-arrestin2/Rap1A interaction is LPA-dependent. The leading edge of motile cells compartmentalizes different protein complexes that orchestrate oriented cell motility [50] . Studies have revealed that the actin scaffolding protein IQGAP1 localizes to the leading edge of motile cells and interacts with actin filaments to cross-link them and thereby regulate cell migration [39]–[41]. β-Arrestins have also been shown to facilitate cell migration by sequestering regulators of actin assembly at the leading edge [4]. We therefore investigated whether the β-arrestin2 was necessary for the recruitment of IQGAP1 to the leading edge of migratory cells.

**Figure 7:**
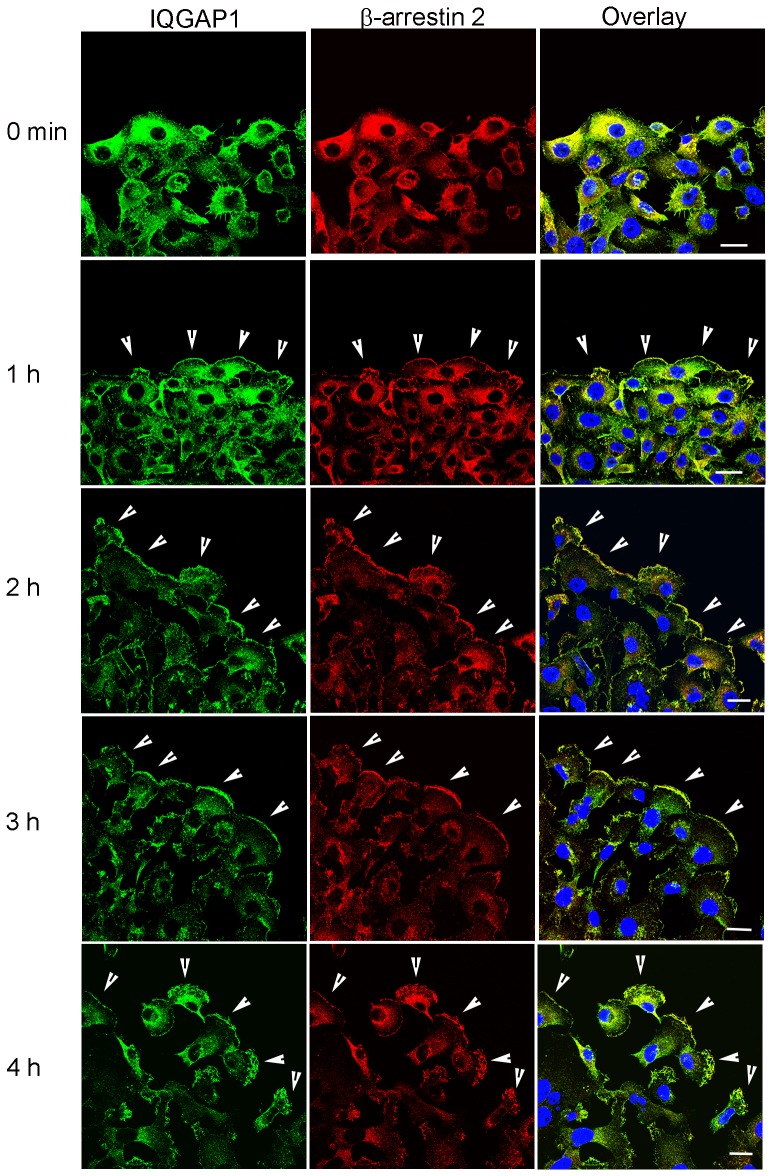
β-Arrestin2 co-localizes with IQGAP1 at the leading edge of migratory breast cancer cells. MDA-MB-231 cells were serum starved for 4h and scratched with a sterile pipette tip. Cells were then placed in serum-free medium containing 10 µM LPA and allowed to migrate into the scratch. Cells were fixed, permeabilized, immunostained and visualized using confocal microscopy. Time course of endogenous IQGAP1 immunostaining performed with rabbit anti-IQGAP1 antibody followed by Alexa-Fluor 488 (green). Endogenous β-arrestin2 immunostaining was performed with a polyclonal goat anti-β-arrestin2 antibody followed by Alexa-Fluor 555 (red). Areas of co-localization in lamellipodia at the leading edge of motile (arrows, overlay) is shown in yellow. Scale bar, 20 µm.

**Figure 8:**
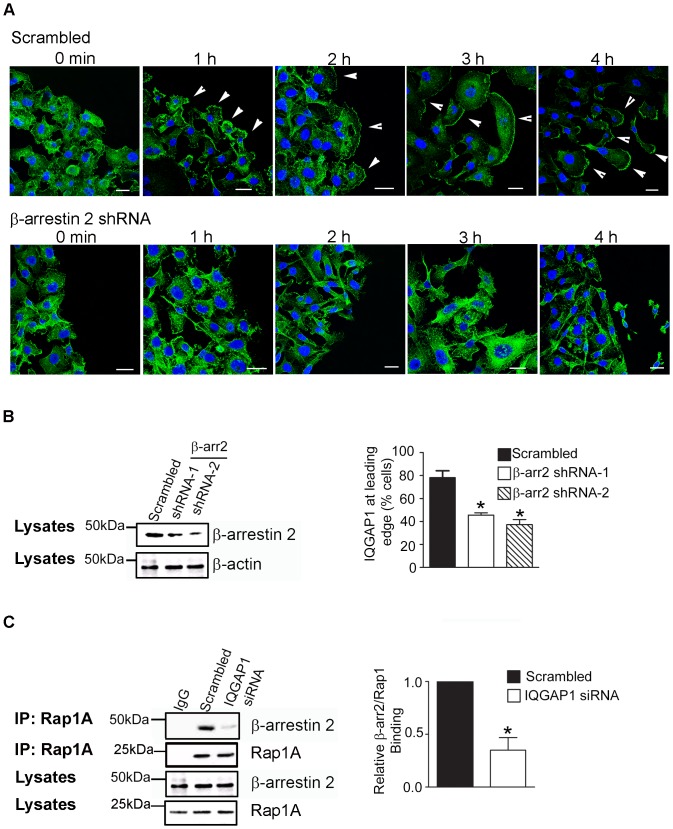
β-arrestin2 regulates LPA-stimulated localization of IQGAP1 to the leading edge. (A) MDA-MB-231 cells were serum starved for 4h and scratched with a sterile pipette tip. Cells were then placed in serum-free medium containing 10 µM LPA and allowed to migrate into the scratch. Cells were fixed, permeabilized, immunostained and visualized using confocal microscopy. LPA (10 µM) stimulates lamellipodia formation and the accumulation of IQGAP1 at the leading edge of motile MDA-MB-231cells (white arrowheads) expressing scrambled shRNA (control, upper panel) in a scratch assay. The effect of depleting endogenous β-arrestin2 from LPA-stimulated cells is shown (lower panel). Endogenous IQGAP1 immunostaining was performed with rabbit anti-IQGAP1 antibody followed by Alexa-Fluor 488 (green). Scale bar, 20 µm. (B) β-arrestin2 expression in MDA-MB-231 cells stably expressing shRNA against β-arrestin2 was verified by Western blot analysis. A representative blot from three independent experiments is shown. Graph showing blind quantification of cells containing IQGAP1 at the leading edge of migrating cells from (A) carried out after stimulation of cells with LPA for 4 hours, as described under “Experimental Procedures”. Error bar, SEM *, p<0.05. Data from three independent experiments. (C) Depletion of IQGAP1 blocks the endogenous association of Rap1A and β-arrestin2 in MDA-MB-231 cells. Co-immunoprecipitation studies were performed using a rabbit polyclonal anti-Rap1A antibody. Endogenous β-arrestin2 was detected with a monoclonal anti-β-arrestin2 antibody. A representative blot from three independent experiments.

We first characterized the sub-cellular localization of β-arrestin2 and IQGAP1 in motile MDA-MB-231 breast cancer cells that were scratched and allowed to migrate in the presence or absence of LPA. In the absence of LPA, we found that β-arrestin2 co-localized with IQGAP1 in cells (Figure 7); this is consistent with our finding that β-arrestin2 and IQGAP1 are found in a complex under basal condition (Figure 5B, C). Upon LPA treatment, β-arrestin2 co-localized with IQGAP1 in lamellipodia and membrane ruffles that were formed at the leading edge of migrating cells (Figure 7). Breast cancer cells stably expressing β-arrestin2 shRNA showed a pronounced reduction of LPA-stimulated lamellipodia formation in cells at the leading edge (Figure 8, lower panel) compared to cells expressing scrambled shRNA (Figure 8, upper panel). Furthermore, we observed less accumulation of endogenous IQGAP1 at the leading edge of cells expressing β-arrestin2 shRNA, compared to scrambled controls (Figure 8A, B). These results reveal that β-arrestin2 is required for the localization of IQGAP1 at the leading edge of motile breast cancer cells and for directional migration.

This far our data indicate that β-arrestin2 scaffolds Rap1A and IQGAP1 under basal conditions, and LPA stimulation further enhances these interactions (Figure 5). Since IQGAP1 binds Rap1A and β-arrestin2 constitutively (Figures 5B and 6, respectively), we therefore sought to determine whether depletion of IQGAP1 from MDA-MB-231 cells impairs Rap1A/β-arrestin2 interactions. Indeed we found a significant loss of binding between these two proteins in cells stably expressing IQGAP1 siRNA, compared to scrambled controls (Figure 8C). Taken together, our results suggest that a complex of β-arrestin2, Rap1A and IQGAP1 regulates LPA induced lamellipodia formation and breast cancer cell migration.

## Discussion

In this study, we are the first to describe a link between LPA_1_ stimulation, β-arrestin2-containing complexes and the activation of Rap1 GTPases that is necessary for the acquisition of a migratory and invasive phenotype. We have identified that Rap1A and the scaffolding protein IQGAP1 are novel binding partners for β-arrestin2 as well as LPA_1_ receptors in breast cancer cells. We found that Rap1A, as well as β-arrestin2 and LPA_1_ [20] are aberrantly expressed in breast cancer cells and patient tumors, and previous studies have shown that IQGAP1 levels are elevated in breast carcinoma [49]. We thus postulate that in breast tumors, LPA_1_ activation recruits β-arrestins and leads to the activation of Rap1, and signaling via IQGAP1. This modulates the actin cytoskeleton, a process necessary for cell migration and invasion. Based on our data, we propose a model for chemoattractant-induced breast cancer cell cytoskeletal reorganziation. Under basal conditions, IQGAP1 scaffolds β-arrestin2 and Rap1A in cells. In response to LPA_1_ activation, β-arrestin2 is recruited to LPA_1_, to mediate LPA_1_ endocytosis [24], LPA enhances the binding of β-arrestin2 to Rap1A which then activates this GTPase. β-Arrestin2 also recruits IQGAP1 to the leading edge of migratory cells. Whether LPA_1_ activation further regulates the level of association between β-arrestin2/IQGAP1 appears to be cell-specific. Although Rap1 has been implicated in growth factor signaling pathways [34], [36], [51], the direct involvement of Rap1A in LPA induction of lamellipodia formation and forward movement is a novel function for this GTPase.

β-Arrestins function as signaling scaffolds facilitate cell migration by sequestering regulators of actin assembly at the leading edge [5], [9]. β-Arrestins associate with cofilin, an actin filament severing protein, localizing it to the leading edge of cells to promote barbed end formation and membrane protrusions [4]. β-Arrestins also interact directly with Arp2/3 complex and WASP family of proteins and can thereby affect actin nucleation [52]. IQGAP1 also serves as a scaffold to integrate multiple signaling molecules to facilitate cell migration [53]. β-Arrestins and IQGAP1 can bind microtubules that control polarized cell motility [41], [54]. We establish that the molecular scaffolds β-arrestins and IQGAP1 associate with each other in breast cancer cells, to perhaps fine tune their signaling capacity. Indeed other reports have described complex formation between scaffolds [55], [56] .

We identified a novel interaction between LPA_1_ and IQGAP1 in MDA-MB-231 cells. IQGAP1 has been shown to be enriched at invadopodia in MDA-MB-231 cells, structures that form at contact sites between invasive tumor cells and the extracellular matrix [57]. Although it is not surprising that knock-down of IQGAP1 disrupted general cell invasion, we found that LPA-induced breast cancer cell invasion specifically required the presence of IQGAP1, thus establishing it as a key player in this process. A recent study reported that β-arrestin2-mediated scaffolding of the RAF-MEK-ERK1/2 signaling components is required for the expression and secretion of MMP-1 by bronchial epithelial cells [58]. Thus, both IQGAP1 and β-arrestins act to scaffold components of the MEK/ERK pathways [39], suggesting that IQGAP1 and β-arrestin2 may utilize the MAPK signaling cascade to regulate LPA-induced breast cancer cell invasiveness.

Rap1 has been shown to elicit both pro- and anti-invasive phenotypes depending on the cancer type [36], [38], [59], [60]. However, few studies have examined a role for Rap1 in breast cancer [38], [61]. Rap1 activity was found to be higher in malignant human T4-2 breast cancer cells compared to the non-malignant S1 cells [38] and expression of constitutively active Rap1 in T4-2 cells yields larger tumors with higher grade of malignancy *in vivo* [38]. Recently, Rap1 has been shown regulate breast cancer cell migration *via* the adhesion molecule JAM-A [62]. We found higher levels of Rap1A mRNA in Stage I–IV breast cancer tumors relative to Stage 0, normal non-diseased tissue samples. This implicates Rap1A in tumorigenesis before the cancer has spread to the nodes (Stage I) as well as during tumor metastasis (Stages II–IV). Correspondingly, we observed that Rap1A protein expression increased in DCIS and invasive tumors in comparison to normal ducts. These findings are in line with recent findings by Furstenau *et al.* that show that Rap1 is weakly expressed in normal and benign breast tissue while DCIS lesions and areas with invasive tumors show enhanced Rap1 expression [37]. We also observed increased levels of Rap1A protein in invasive breast cancer cells (MDA-MB-231 and Hs578T) compared to non-invasive MCF-7 cells or the non-malignant MCF-10A cells. Overall, these results indicate that the activity or function of Rap1A may be altered in aggressive cancer cells. Indeed, LPA only induced Rap1 activity in the highly metastatic MDA-MB-231 cells and not in the non-malignant MCF-10A cells. These results suggest that chemoattractants such as LPA may selectively stimulate Rap1 in tumor cells.

We identified a novel interaction between LPA_1_ and Rap1A in breast cancer cells. MDA-MB-231 cells primarily express LPA_1_ mRNA [20]. Studies looking at endogenous LPA receptors could not be conducted due to lack of effective antibodies against LPA receptors. The binding of Rap1A to LPA_1_ might stimulate signaling pathways involved in cell motility and invasion. Reduction of Rap1A expression using shRNA sequences inhibited LPA-stimulated breast cancer cell migration and formation of invasive stellate structures in 3D cultures. These 3D tumor invasion models provide important insights into breast cancer biology including cellular differentiation and tissue organization [20]. Although the specific mechanisms by which Rap1A mediates cell invasion are unclear, Rap1A has been shown to stimulate MMP-7 gene transcription in squamous cell carcinoma cells via β-catenin [35]. The possible role of Rap1 in the expression of genes involved in breast cancer invasion is currently under investigation.

In summary, our data indicate that β-arrestin2 regulates multiple and distinct, yet convergent, signaling pathways to modulate the cytoskeletal reorganization necessary for breast cancer cell migration and invasion. Whether the β-arrestin2/Rap1A/IQGAP1 pathway regulates cell migration downstream of other GPCRs remains to be tested. Since β-arrestins regulate signaling of several types of receptor systems, a better understanding of the molecular pathways by which β-arrestins regulate breast cancer invasiveness will aid in the identification of new targets for the design of novel therapeutics against metastatic disease.

## Materials and Methods

### Cell culture

Cell lines (MCF-10A, MCF-7, MDA-MB-231 and Hs578T) were from American Type Culture Collection (ATCC) and grown as described [20]. Cells were directly obtained from and characterized by ATCC as per Cell Line Verification Test Recommendations (ATCC Technical Bulletin No. 8). All cells were passaged in our laboratory for a maximum of two months.

### Reagents and constructs

LPA (18:1) is from Avanti Polar Lipids. YFP-Rap1A and myc-Rap1A are generous gifts from Dr. Mina Bissell (Berkeley, CA) and Dr. Johannes Bos (Utrecht, Netherlands), respectively. FLAG-LPA_1_ has been described [20]. pSuper-IQGAP1 siRNA were generously provided by Dr. David Sacks (Bethesda, MD). Rap1A-pRS-shRNA is from Origene Technologies.

### Quantitative real-time PCR

Experiments were done as described [20]. A cDNA array generated from breast cancer tissue (Stages 0-IV) was purchased from Origene Technologies. Stage 0 samples were derived from tissues that were determined to be normal, as determined by a pathologist (www.origene.com). The sequences for Rap1A primers are 5`-ACAGGACCTGAGGGAACAGA-3` and 5`-CCCTGCTCTTTGCCAACTAC-3`.

### Tissue staining and confocal microscopy

Formalin-fixed and paraffin-embedded human normal breast tissue, DCIS, and invasive cancer tissue sections (5 µm thick) were obtained from London Health Sciences Center and characterized by a pathologist on our team (Dr. Siddiqui). These studies were conducted in consultation with a pathologist on our team (Dr. Siddiqui). Serial sections were de-paraffinized, cleared, rehydrated through an ethanol series, and subjected to either hematoxylin and eosin staining or immunofluorescence (IF) using polyclonal anti-Rap1A rabbit antibody (1:50, Santa Cruz), polyclonal anti-β-arrestin2 goat antibody (1:50, C-18, Santa Cruz) and monoclonal anti-CAM5.2 mouse antibody (undiluted, BD Biosciences). Tissue sections were imaged using Zeiss LSM-510 META laser scanning microscope (Zeiss).

### Stable transfections and mRNA silencing

MCF-10A and MDA-MB-231 cells were electroporated with 25 µg of DNA using Gene Pulser Xcell (Bio-Rad) as described [20]. Heterogeneous populations of cells transfected with YFP-Rap1A or FLAG-LPA_1_ were selected using G418. Generation of MDA-MB-231 cells stably expressing β-arrestin 2 shRNA constructs have been previously described [20]. To knockdown Rap1A, four different Rap1A shRNA were individually electroporated into MDA-MB-231 cells. The two constructs that gave the best knockdown were selected and a heterogeneous population of cells expressing each Rap1A shRNA construct was used for subsequent studies (shRNA-1, GCCAACAGTGTATGCTCGAAATCCTGGAT; shRNA-2, CTATTACAGCTCA-GTCCACGTTTAACGAC).SiIQGAP1-pSuperRenilla [40] was microporated (Bio-Rad) according to the manufacturer’s instructions.

### Co-immunoprecipitation and immunoblots

Rap1A expression in cell lines was examined using a rabbit anti-Rap1A antibody (1:500, Santa Cruz). Equal loading of the samples was verified by checking β-actin expression (Rabbit antibody, 1:2000, Sigma Aldrich). Co-immunoprecipitation experiments between endogenous proteins were conducted as previously described [20]. To investigate the interaction between LPA_1_ receptor and YFP-Rap1A, MDA-MB-231 FLAG-LPA_1_ stables [20] were stably transfected with YFP-Rap1A using Lipofectamine 2000 (Invitrogen) and cell lysates subjected to co-immunoprecipitation assays.

### Rap1 activation assay

A Rap1-activation assay was performed according to the manufacturer’s instructions (Upstate Millipore). Cells were serum-starved for 4h and active Rap1 was pulled down from lysates using agarose beads conjugated to RalGDS-RBD and detected by Western blot analysis using a rabbit anti-Rap1 antibody (Upstate).

### Immunostaining

Assays were conducted as previously described [20]. We employed rabbit anti-IQGAP1 (1:500, Santa Cruz) and goat anti-β-arrestin2 (C-18, 1:50, Santa Cruz) primary antibodies followed by anti-rabbit AlexaFluor-488 (1:250, Molecular Probes) and anti-goat AlexaFluor555 (1:1000, Molecular Probes) secondary antibodies. Nuclei were labeled using Hoechst 33258 (1:10000, Invitrogen). All images were taken using Zeiss LSM-510 META laser scanning microscope (Zeiss).

### 3D invasion assays

3D invasion assays were conducted as described [20]. Cells were plated in a 1:1 dilution of phenol red-free Matrigel and culture medium containing 2.5×10^6^ cells/mL on Matrigel-coated 35 mm glass-bottomed culture dishes (MatTek). Cell colonies were scored in a blinded manner as either stellate or spheroidal. Stellate colonies were characterized by one or more projections from the central sphere of cells. Images were taken on an IX-81 microscope (Olympus) using InVivo Analyzer Suite (Media Cybernetics).

### Cell migration and invasion assays

Transwell chamber migration and Matrigel-invasion assays (Millipore filters, 8 µm pores) were performed as detailed [20]. Serum-starved (4h) cells (40,000) were placed in the upper chamber and allowed to migrate for 18 hours. Ten fields of nuclei images were acquired and quantified (20x magnification). Migration was expressed as a percentage of control cells. We typically observed approximately 200 cells migrated per field.

### Cell adhesion assays

Quantitative cell adhesion assays were carried out as described [63], using non-tissue culture-treated 96-well plates coated with 10 mg/ml fibrnectin for 1h, at room temperature. Briefly, fibronectin coated and uncoated control wells were blocked for 1 h with 5% bovine serum albumin (BSA). Cells were split one day prior to the experiment to achieve a subconfluent culture. Briefly, serum-starved cells (2X10^4^) were added to each well and incubated for 60 min at 37°C. Each experiment was done in triplicate. Unattached cells were removed and stained with trypan blue to exclude the possibility of lack of adhesion, due to cell death. Wells were washed three times with PBS and to exclude non-specific adhesion. Attached cells were fixed in 3.7% formaldehyde, rinsed with PBS, and stained with 1% crystal violet in 2% ethanol, lysed in 10% acetic acid and OD_600_ was determined in a plate reader.

### Scratch assays for cell motility

Serum-starved (4h) cells were scratched and allowed to migrate for 16 hours. Time-lapse images and movies of scratch closure were collected every 10 minutes using an IX-81 inverted microscope (Olympus). Distance travelled (in µm) was measured using ImagePro software (Media Cybernetics). Experiments were done in duplicate. The quantitation of the number of cells containing proteins at the leading edge of the cell was carried out using 100 cells/condition from three experiments. Briefly, a cell containing a positive immunofluorescence signal only along the edge of the plasma membrane that was directly adjacent to the scratch was scored as positive for leading edge localization. If a cell contained signal along the complete cell periphery, it was scored as negative for leading edge staining.

### Statistical Analysis

Student's t test or one-way ANOVA was used to assess statistical significance with GraphPad Prism 5.0 software (GraphPad Software, Inc.). Differences with p < 0.05 were considered significant.

## Supporting Information

Figure S1 **Knockdown of Rap1A by shRNA does not affect cell viability.**MDA-MB-231 cells stably expressing scrambled shRNA or Rap1A shRNA were subjected to MTT cell viability assays. Absorbance was measured at 575 nm and a reference reading at 750 nm. Columns represent mean absorbance from three independent experiments.
(TIF)

Figure S2 **Effect of Rap1A depletion on MDA-MB-231 cell adhesion.** MDA-MB-231 cells stably expressing scrambled shRNA or Rap1A shRNA were subjected to adhesion assays to fibronectin. Columns show % adhesion compared to scrambled control; data from four independent experiments.
(TIF)

Movie S1 **Knockdown of Rap1A by shRNA inhibits motility of MDA-MB-231 cells by scratch assay.** The top panel of the video shows live imaging of cells expressing a scrambled control while the bottom portion shows cells knocked down for Rap1A. Serum-starved cells were stimulated with 10 µM LPA and live-cell microscopy conducted using an IX-81 microscope once every 10 min over a course of 16 hours and compiled into a time-lapse video. *Top panel,* cells expressing scrambled shRNA control; *bottom panel,* cells expressing Rap1A shRNA.
(MOV)
