# Supplementary figures and images for: β-Arrestin2 Regulates Lysophosphatidic Acid-Induced Human Breast Tumor Cell Migration and Invasion via Rap1 and IQGAP1
Source: PLoS One. 2013 Feb 6;8(2):e56174. doi: 10.1371/journal.pone.0056174 (PMC3566084; doi:10.1371/journal.pone.0056174)

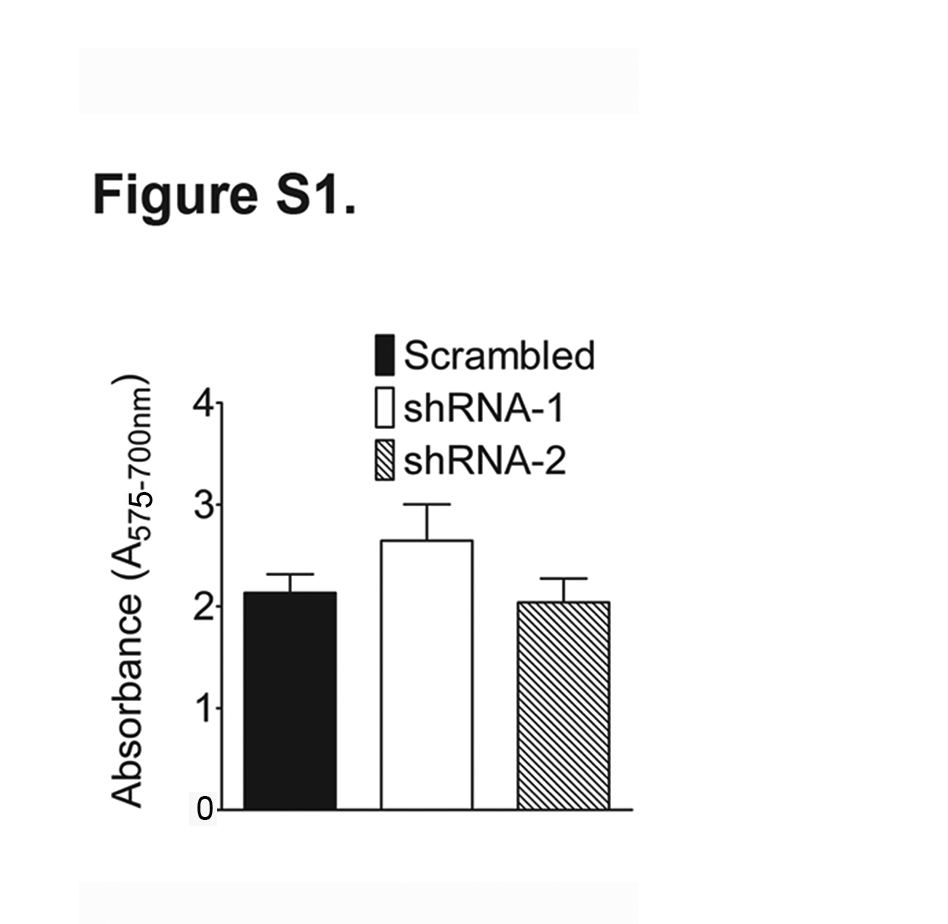

Supplement: Figure S1 — Knockdown of Rap1A by shRNA does not affect cell viability.MDA-MB-231 cells stably expressing scrambled shRNA or Rap1A shRNA were subjected to MTT cell viability assays. Absorbance was measured at 575 nm and a reference reading at 750 nm. Columns represent mean absorbance from three independent experiments. (TIF) [file pone.0056174.s001.tif]

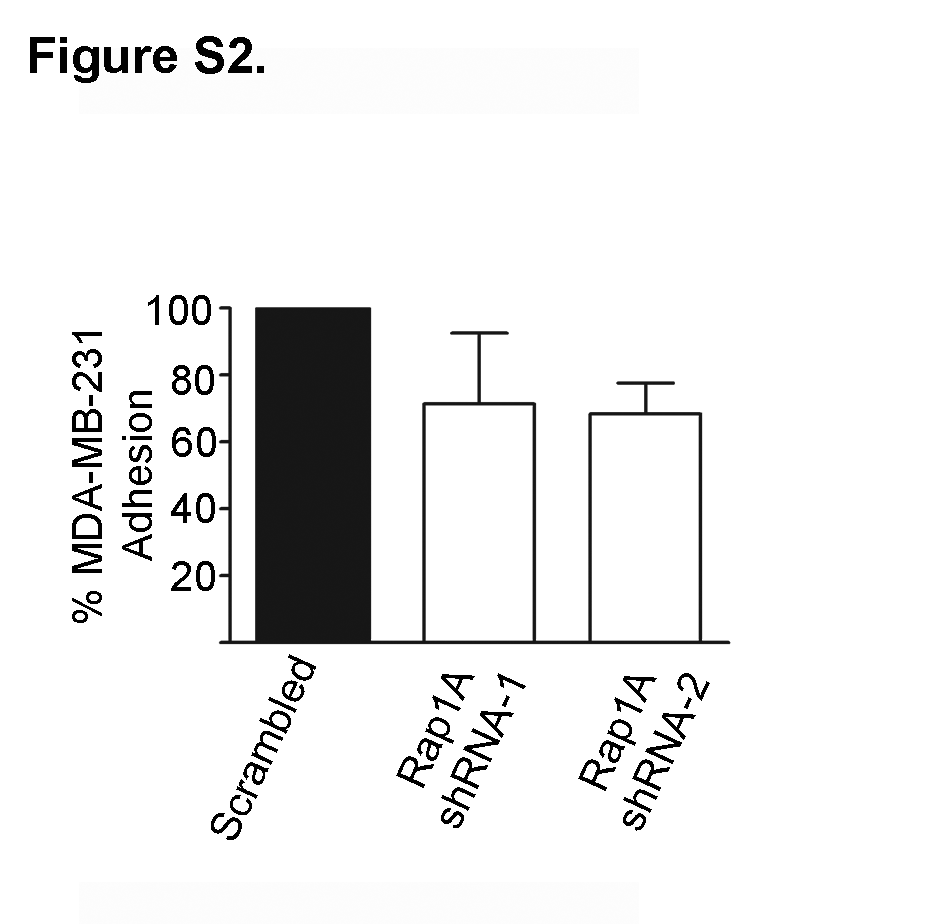

Supplement: Figure S2 — Effect of Rap1A depletion on MDA-MB-231 cell adhesion. MDA-MB-231 cells stably expressing scrambled shRNA or Rap1A shRNA were subjected to adhesion assays to fibronectin. Columns show % adhesion compared to scrambled control; data from four independent experiments. (TIF) [file pone.0056174.s002.tif]
